# CCR5 activation and endocytosis in circulating tumor-derived cells isolated from the blood of breast cancer patients provide information about clinical outcome

**DOI:** 10.1186/s13058-022-01528-w

**Published:** 2022-05-23

**Authors:** Ashvathi Raghavakaimal, Massimo Cristofanilli, Cha-Mei Tang, R. K. Alpaugh, Kirby P. Gardner, Saranya Chumsri, Daniel L. Adams

**Affiliations:** 1 University of Pennsylvania, Philadelphia, PA 19104 USA; 2 Robert H Lurie Cancer Center, Northwestern University, Chicago, IL 60611 USA; 3 Creatv MicroTech, Inc., Rockville, MD 20850 USA; 4 Fox Chase Cancer Center, Philadelphia, PA 19111 USA; 5 Rutgers, The State University of New Jersey, New Brunswick, NJ 08901 USA; 6 Creatv MicroTech, Inc., Monmouth Junction, South Brunswick, NJ 08852 USA; 7 Mayo Clinic Cancer Center, Jacksonville, FL USA

**Keywords:** Breast cancer, Liquid biopsy, Circulating tumor cells, CCR5, Metastasis

## Abstract

**Background:**

CCR5 is a motility chemokine receptor implicated in tumor progression, whose activation and subsequent endocytosis may identify highly aggressive breast cancer cell subtypes likely to spread into the circulatory system.

**Methods:**

The MDA-MB-231 cell line was used to model and visualize CCR5 activation by stimulation with RANTES, in an effort to quantify CCR5 endocytosis from the cell surface to the perinuclear space. CCR5 expression was then examined in tumor-associated cells (TACs), consisting of circulating tumor cells and circulating stromal cells, isolated from the peripheral blood of 54 metastatic breast cancer (mBC) patients to evaluate these CCR5 pooling patterns as they relate to progression and survival over 2 years.

**Results:**

In MB231 experiments, it was observed that CCR5 formed ~ 1 micron clusters identified as “CCR5 pools” on the surface of the cell, which in the presence of RANTES were endocytosed and translocated to the cell cytoplasm. When TACs from patients were analyzed, CCR5 pools were observed on the cell surface and translocating to the nuclear area, with CCR5 also having a positive statistical correlation between increased numbers of TACs and increased CCR5 pools on the cells. Further, it was determined that patients with very high numbers of CCR5 (> 10 CCR5 pools), specifically in the circulating stromal cells, were associated with worse progression-free survival (hazard ratio = 4.5, *p* = 0.002) and worse overall survival (hazard ratio = 3.7, *p* = 0.014).

**Conclusions:**

Using a liquid biopsy approach, we evaluated two populations of tumor-associated cells emanating from primary tumors, with data suggesting that upregulation of the motility chemokine CCR5 in TACs provides clinically relevant opportunities for treating and tracking drug targetable receptors in mBC.

**Supplementary Information:**

The online version contains supplementary material available at 10.1186/s13058-022-01528-w.

## Introduction

Breast cancer (BC) is the leading cause of cancer in the USA. In 2021, SEER estimates 284,200 new cases of BC with 44,130 deaths attributed to the disease [1]. The C–C chemokine receptor type 5 (CCR5), a motility marker, has been implicated in the regulation of tumor progression and metastatic spread in BC [2]. CCR5 has been found to be upregulated in aggressive breast cancer and may lead to cancer cell honing to metastatic sites [3]. CCR5 signal is found to be low to nonexistent in primary BC tumors but upregulated in secondary sites of metastasis [2]. CCR5 regulates cancer cell migration, which indicates that CCR5 + cells may be more migratory in nature and may promote metastasis of disease[4]. This has been shown by elevated CCR5 signal being associated with increased tumor progression in highly invasive BC subtypes (i.e., basal and HER-2) [3, 5–7]. Furthermore, upregulated CCR5 signaling has been positively correlated with axillary lymph node metastasis which supports the concept that CCR5 is associated with more aggressive disease spread [7, 8]. As a result of this correlation, there is an interest in studying the role of upregulation in the CCR5 pathway, as evidenced by a number of clinical trials targeting CCR5 as a point of inhibition to possibly reduce the motility of cells spreading BC [3, 9–12].

**Fig. 1:**
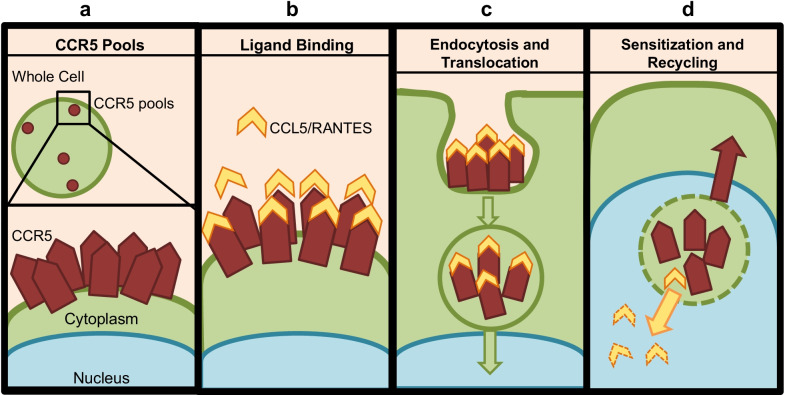
Diagram of the Activation and Endocytosis of CCR5. **a** CCR5 pools exist on the surface of the cell. The pools are a grouping of multiple aggregates of colocalized CCR5 receptors (red pentagons). **b** The ligand RANTES (yellow chevron) binds to and activates the CCR5. **c** Activated CCR5 pools are endocytosed and then translocated to the perinuclear space. **d** Once in the perinuclear space, CCR5 receptors are resensitized and recycled back to the cell surface as RANTES is degraded

The CCR5 receptor and its associated pathway are activated through a number of ligands (i.e., CCL3, CCL5, CCL8, etc.), the primary being chemokine C–C Ligand 5 (CCL5) (e.g., RANTES) [6, 9–12]. RANTES activates CCR5 allowing BC cells to enhance their motility, invasiveness, and metastatic potential [13]. The CCR5/RANTES axis and the activation pathway of CCR5 have been studied in a number of model cancer cell lines, including the breast cancer cell line MDA-MB-231 [6]. In MDA-MB-231 cells, it was shown that CCR5 receptors appear as aggregates of dense ~ 1 µm clusters, defined as “CCR5 pools” [2, 14, 15]. The activation of CCR5 via RANTES leads to the endocytosis of these pools, which is a type of receptor-mediated endocytosis resulting in the internalization of the receptor–ligand complex from the cell surface and subsequent translocation to the perinuclear space via an endosome (Fig. 1) [16]. At the perinuclear space, the ligand is disassociated, and the receptor is resensitized allowing the CCR5 to be recycled back to the cell surface (Fig. 1) [16–18]. The formation of CCR5 pools and subsequent endocytosis into the cells are indications of activation of the CCR5 motility pathway [16].

The CCR5/RANTES axis has been shown to help cancer cells recruit monocytes and stromal cells in circulation and “educate” them to take part in the immunosuppressive tumor microenvironment (TME) [9]. For example, CCR5 has recently been found on circulating tumor cells (CTCs) and is implicated in the activation and migration of CTCs from primary tumor sites into circulation [19–21]. It has been shown that macrophages drive tumor cell migration from the primary tumor site to blood vessel and aid intravasation into the blood stream (i.e., becoming CTCs), via a process defined as transendothelial migration [22–28]. It has been demonstrated that tumors recruit macrophages to produce a pro-tumorgenic microenvironment that downregulate the anti-tumor immune response and upregulates tumor growth [3, 9, 23]. Recently, pro-tumorigenic macrophages defined as cancer-associated macrophage-like cells (CAMLs) were found to be bound to CTCs in circulation in 10% of patients [22, 24]. These macrophage-like cells were shown to be present in metastatic BC patients [22, 24] as phagocytic giant polyploid immune cells with protumorigenic characteristics of myeloid lineage. Although CCR5 signaling is not high in primary BC tumor sites, it could be theorized that CCR5 as a motility marker would be upregulated in migratory tumor-associated cells (TACs—CTCs and CAMLs) in circulation which could explain their motility [2, 26–28]. We theorize that if similar patterns of CCR5 signaling can be found in both cancer model cell lines and BC patients, then upregulation and localization of CCR5 in patients may offer novel clinical prognostic applications.

In this study, a CCR5 activation bioassay was developed in the BC cell line MDA-MB-231 to model CCR5 signaling patterns. The MDA-MB-231 cell line was chosen based on prior research studying CCR5 and the ability of MDA-MB-231 cells to form both single-nucleus tumor cells and polyploid giant cancer cells (PGCCs) that can act as a theoretical model for both CTCs and CAMLs [22, 29–31]. As a result, the MDA-MB-231 cell line can model CCR5 signaling patterns within both normal ploidy and hyperploidy cell types before screening in patient samples from the peripheral blood. Here, we observed that CCR5 pools, ligand-induced upregulation of CCR5, and the endocytic internalization pathway appear conserved in both MDA-MB-231 cells and circulating TACs. In patients, we visualized CCR5 pools translocating to the perinuclear space and observed that the increased CCR5 pooling is a clinically significant parameter that stratifies patients based on survival.

## Materials and methods

### Cell line work

MDA-MB-231 metastatic BC cell lines were procured from ATCC (RRID: CVCL_0062). Cells were cultured in Leibovitz’s L-15 media with 10% FBS and 1 × Anti-anti under atmospheric conditions at 37 °C and grown until confluency. Prior to the assay, confluent cells were trypsinized and large PGCC cells (Large Cells) (> 30 µm) were separated from the small cells (< 30 µm) using a size exclusion 30-µm mesh cell strainer (Miltenyi Biotec)^30^. Cells retained on the mesh were > 30 µm, and cells that passed through the filter into a collection flask were < 30 µm in size. Large cells were rinsed off the size-exclusion mesh with media and seeded at ~ 0.5 × 10^6^ cells/well in an 8-well reaction plate (Nunc) for 24 h (Additional file 1: Fig. S1a-c). Small cells were taken from the collection flask, transferred with media, and seeded at ~ 10^6^ cells/ml into an 8-well reaction culture wells (Nunc) and incubated in media for 24 h **(**Additional file 1: Fig. S1d-f). The size-separated populations retained their differential size phenotype after being cultured separately.

MCF-7, Hs 578 T, and SKBR3 were used in the CCR5 bioassay in the absence of RANTES. MCF-7 cell line was procured from ATCC (RRID: CVCL_0031) and cultured in EMEM medium with 10% FBS and 1 × Anti-anti and incubated at 37 °C with 5% ${\text{CO}}_{2}$. Hs 578 T were procured from ATCC (RRID: CVCL_0332) and cultured in DMEM medium with 10% FBS and 1 × Anti-anti and incubated at 37 °C with 5% ${\text{CO}}_{2}$. SK-BR-3 were procured from ATCC (RRID: CVCL_0033) and cultured in McCoy’s 5a medium with 10% FBS and 1 × Anti-anti and incubated at 37 °C with 5% ${\text{CO}}_{2}$.

## RANTES

A solution of 0.1 mg/mL RANTES (PeproTech Inc.) was tagged with a DyLight 650 dye using a DyLight 650 conjugation kit (Thermo Fisher Scientific) and a separate solution of RANTES remained untagged. 2µL of tagged RANTES stock solution at a concentration 12.5 mM was aliquoted into 200µL of media in a well with large cells, and 2µL of untagged RANTES at 12.5 mM was aliquoted into 200µL of media in a well with large cells (Additional file 1: Fig. S1), for both to achieve a concentration of 125 nM RANTES in solution. Likewise, 2µL of tagged RANTES and 2µL of untagged RANTES were aliquoted into two wells with 200µL of media and small cells (Additional file 1: Fig. S1). The control wells, one well of large cells and one well of small cells, did not receive any RANTES (plate layout can be found in Additional file 1: Fig. S1). Cells were incubated in their solution of media for 30 min. The media was then aspirated and the cells were washed with PBS. Cells were dehydrated for 5 min before being fixed with 1X 1% Formaldehyde Fixation Solution (Thermo Fisher) and 1X Triton-X100 Permeabilization Buffer (Thermo Fisher). Plates were washed with 1X PBS and stained for 1 h at room temp with a CCR5 antibody tagged with Dylight 555 (Novus Biologicals Catalog # NBP2-77515R) and LAMP1 antibody (Cell Signaling, clone # D2D11). Plates were washed with 1X PBS with 0.1% Tween-20 and then incubated with FITC tagged anti-rabbit secondary (Cell Signaling, catalog # 4412S) for 30 min. Cells were washed with a 1X PBS with 0.1% Tween solution and PBS and then mounted with DAPI and visualized. This bioassay was conducted in duplicate for this study.

## Primary tumor biopsy

Of the total *n* =54 patients, we received the peripheral blood sample from, only n=15 patients had paired primary tumor biopsy samples available. All available primary tumor FFPE biopsy samples (*n*=15 patients) were obtained through collaboration with Fox Chase Cancer Center in accordance with local IRB regulations and with the written informed consent of patients. Samples were procured at time of surgery from primary breast cancer, stained, and analyzed for CCR5 signal at Fox Chase Cancer Center.

## Blood sample collection

Anonymized de-identified peripheral blood samples from 54 patients with confirmed mBC over 18 years of age were obtained. mBC patients were obtained through collaboration with University of Maryland Baltimore, Fox Chase Cancer Center, and Northwestern University all in accordance with local IRB regulations and with the written informed consent of all patients across multiple metastatic breast cancer pilot studies (Table 1). All samples were procured at time of scan confirming progressive metastatic disease by RECIST criteria, and these patients had failed at least one line of therapy prior to this study. Prior therapies are defined as the lines of treatment in the current metastatic breast cancer setting. Blood samples were collected and anonymized in a CellSave™ preservative vacutainer (Menarini Silicon-Biosystems) as previously described[22, 25, 29, 32–34].

**Table 1:** Patient Demographic Data A total of 54 metastatic breast cancer patients were recruited for this study

		(*n* = 54)
Age (median)		57 years
Gender	Female	54 (100%)
Race	Asian	4 (7%)
	Black	4 (7%)
	Hispanic	1 (2%)
	White	33 (61%)
	Other/unknown	12 (22%)
Grade	0	1 (2%)
	1	1 (2%)
	2	14 (26%)
	3	21 (39%)
	Unknown	17 (31%)
Histology	IDC*	48 (76%)
	ILC	3 (5%)
	Unknown	12 (19%)
Estrogen receptor	Positive( +)	22 (41%)
	Negative (−)	27 (50%)
	Unknown	5 (9%)
Progesterone receptor	Positive ( +)	13 (24%)
	Negative (-)	37 (69%)
	Unknown	4 (7%)
HER2 receptor	Positive ( +)	11 (20%)
	Negative (−)	40 (74%)
	Unknown	3 (6%)
TNBC		20 (37%)
CAMLs present		53 (98%)
Average CAMLs per patient	5 CAMLs	
CTCs present		34 (63%)
Average CTCs per patient		4 CTCs
Average prior therapies		3

Number of average prior therapies was calculated using patients with a confirmed number of prior therapies. *9 IDC patients were of the IBC subtype

## LifeTracDx microfiltration procedure

Blood samples were shipped overnight at ambient temperature to Creatv MicroTech’s New Jersey laboratory and run using LifeTracDx Microfiltration Assay with a standard of 7.5 ml of peripheral blood through a low-pressure ~ 15 mbar vacuum system, as previously described [22, 25]. CTCs and CAMLs were isolated by size exclusion and then visualized by their expression of cytokeratin labeled by FITC and CD45/CD14 labeled by Cy5 antibodies (prediluted LifeTracDx antibody solution) [22, 25, 29, 32, 33, 35, 36]. Peripheral blood samples were prefixed for 15 min and then filtered through a CellSieve microfilter using a vacuum filtration (~ 3 min). Filters were then washed with 6 ml PBS, placed in postfixation buffer for 15 min and permeabilization buffer for another 15 min. The cells on the filter were stained with the antibody mixture, washed, and mounted with Fluoromount-G with DAPI (Southern Biotech). All cells were imaged, and cell populations were identified as previously described [33]. CTCs were identified by the parameters of being negative for CD45 and positive for cytokeratin and DAPI. CAMLs were identified by presence of CD45/CD14 as macrophages, a cell size ranging from 30 to 300 µm with some showing cytokeratin positivity, and an enlarged nuclear region ranging from 14 to 64 µm in diameter with multiple nuclei in one cell [25, 33–35, 37]. After cell identification, filters were then quenched to remove fluorescent signal, as previously described [32, 38] and re-stained with CCR5-Dylight555. Following an hour of staining, the filters were washed with 10 ml PBS + 0.1% Tween-20 (PBST), 3 ml PBS, placed onto a microscope slide, and mounted with Fluoromount-G with DAPI (Southern Biotech). All previously identified cells were then reimaged with the CCR5-Dylight555. In total, peripheral blood samples were all prospectively procured from patients to evaluate CCR5 staining, shipped, and processed within 96 h, as previously described. Blood filtration, cell staining, and cell mounting were completed within 6 h of processing and placed at 4C until image analysis. Cells were enumerated and imaged within 10 days of processing. Direct CCR5 staining without quenching was done on *n* = 23 samples averaging 13 CCR5 pools/cell. Quenching and follow-up staining with CCR5 were done on *n* = 29 samples within 6 months of initial processing after storage at 4C, averaging 15 CCR5 pools/cell. CCR5 pooling was equally quantifiable in both methodologies.

## Quantifying intensity of CCR5 signal

Immunofluorescence-stained CCR5 and RANTES were visualized on cells by an Olympus BX54WI Fluorescent microscope with a Carl Zeiss AxioCam. A Zen 2011 Blue Edition (Carl Zeiss) was used to process the images. In cell lines, CCR5 signal was calculated by outlining the perimeter of the cell of interest and calculating the intensity of the signal. To measure nuclear CCR5 signal, CCR5 signal was outlined on the border of the DAPI-stained nuclear region of the cell of interest [22, 32, 38]. CCR5 signal was considered negative if the intensity mean value (calculated by Zen Blue Image Processing) was below 250, low if the value was between 251 and 1000, and high if the value was above 1000. These thresholds were determined based on the fluorescent dye, filter cube, and exposure used [22, 32, 38].

In patient samples, the CCR5 pools were manually counted using Zen Blue software on each cell of interest (CTCs, CAMLs, etc.) in order to quantify signal intensity. CTCs and CAMLs were found independent of one another and were evaluated independently for CCR5 signal.

## Statistics and analysis

Box-and-whisker plots and Wilcoxon rank-sum tests were run on MATLAB 2020b. Statistical analysis results for ANOVA and log rank analysis for Kaplan–Meiers were run using MATLAB 2020b with a p-value of less than 0.05 being considered significant [39]. Multivariate Cox proportional hazards analysis was run on MATLAB 2020b with a threshold of *p* ≤ 0.05 using all available clinical parameters. For univariate and multivariate analysis, CCR5 expression on CAMLs, lymph node spread, brain metastases, non-brain/lung/liver metastases, hormone status were all run as binary variables; age, CAML number, and CTC number were run as continuous variables; histology, number of organs involved in metastasis, and grade were run as categorical variables. Progression-free survival (PFS) was defined at the time of blood draw to progression of disease as defined by RECIST criteria. Overall Survival (OS) was defined as time of blood draw to death of the patient. Follow-up clinical data were available for 50 of the 54 recruited patients, with 4 patients having no follow-up progression/death information, and were not included in survival analysis.

## Results

### CCR5 in cell lines RANTES upregulation and CCR5 pools

**Fig. 2:**
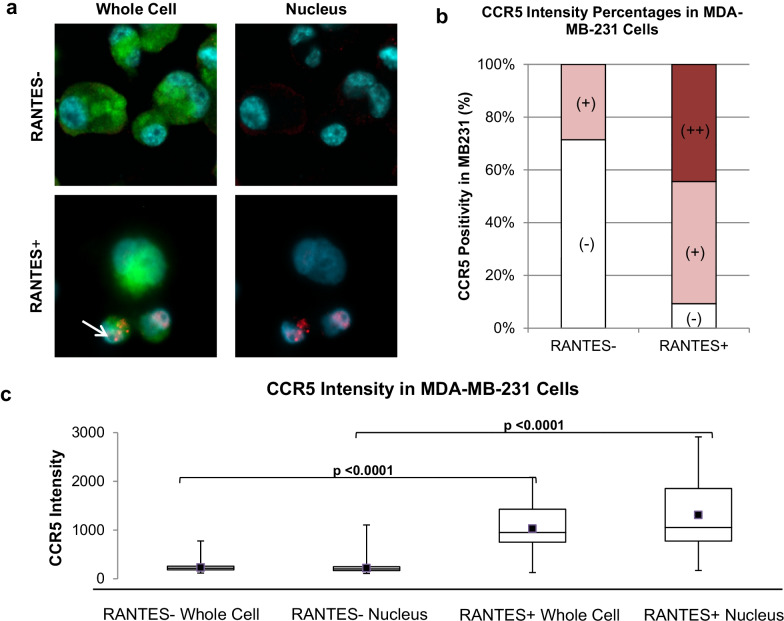
CCR5 Activation by RANTES in Model MDA-MB-231 Cell Line. **a** MB231 cells show overlapping cytoplasm (green) and nucleus (light blue), with or without CCR5 signal (red). MB231 cells without the addition of RANTES are devoid of CCR5 (top panels). MB231 with the addition of RANTES shows CCR5 positivity (bottom panels), including CCR5 pools (white arrow). **b** In cells not exposed to RANTES, a majority of cells (~ 71%) are CCR5 (−) and 29% of cells being low CCR5 expressing ( +). In contrast, after exposure to RANTES, 9% of cells remained CCR5 (−), with the majority (~ 91%) being medium or high expressing for CCR5. **c** Cells not exposed to RANTES had low CCR5 expression on the surface and within the nucleus. In contrast, the addition of RANTES had high expression/upregulation of CCR5 signal on the cell and within the nucleus

We validated the methods developed by Signoret et al. and Velasco-Velazquez et al. using MDA-MB-231 cell line as a triple-negative mBC model system to visualize low and high CCR5 signals, as well as CCR5 pool activation and internalization via endosomes in the cells (Fig. 2) [6, 7, 13, 16]. RANTES- MDA-MB-231 cells had lower overall CCR5 expression, with 71% of cells found to be CCR5 negative with the remaining 29% showing low expression (Fig. 2). Interestingly, RANTES + MDA-MB-231 cells had a distinctly higher expression of CCR5 (44% high expression, 46% low expression, and 9% negative) as well as increased CCR5 internalization (Fig. 2b). The drastic upregulation CCR5 with the addition of RANTES confirms previous findings and identifies the activation pathway of CCR5 via RANTES in MDA-MB-231 cell lines [6, 7]. In addition to the upregulation and nuclear translocation of CCR5, we visualized the expression of CCR5 pools as previously described by Signoret et al., which appeared as distinct dots in and on the cells (Fig. 2a) [16]. While it has been established that CCR5 internalizes in response to RANTES, we looked to confirm the internalization pathway of CCR5 by staining cells with the endosome marker lysosome-associated membrane protein 1 (LAMP1) in order to visualize the co-localization of CCR5 and RANTES in endosomes (Additional file 1: Fig. S2). We used both tagged and untagged RANTES to ensure that the tag did not negatively interfere with the activation of CCR5. While endosomes are non-specific to the CCR5 pathway and occur abundantly in cells, we identified numerous instances of co-localized overlapping of LAMP1, CCR5, and tagged RANTES signal (Additional file 1: Fig. S2). We then stained patient blood sample TACs with LAMP1 and CCR5, visualizing the conserved pattern of CCR5 internalization through an endosome (Additional file 1: Fig. S2). This appeared to confirm that CCR5/RANTES co-localization is found in endosomes, confirmed by LAMP1 positivity. Interestingly, the MDA-MB-231 cell line produces both single ploidy and hyperploidy cells, previously described as PGCCs [30]. Further, our group has previously found, both single ploidy tumor cells (i.e., CTCs) and enlarged polyploid cells (i.e., CAMLs) that exist in the circulation of breast cancer patients [22, 29, 33]. We choose to examine large, polynucleated MDA-MB-231 cells to see if any unique patterns of CCR5 signaling (for example, increased activation, more surface signal, or more internalized signal) existed in those cells versus the normal-sized single nucleated MDA-MB-231 cells. Because no model cell line exists for CAMLs, we chose to test this hypothesis in MDA-MB-231 cells, as they are known to produce hyperploidy giant cells. We hypothesized that if larger polynucleated cells presented any unique CCR5 patterns, these patterns may be conserved between polynucleated MDA-MB-231 cells and polynucleated CAMLs from patients**.** However, it was determined that there was no quantifiable difference between different ploidy populations (Additional file 1: Fig. S3), which allows for large ploidy cells and small cells to be treated as one population in downstream CCR5 activation experiments.

## CCR5 in breast cancer patient TACs

**Fig. 3:**
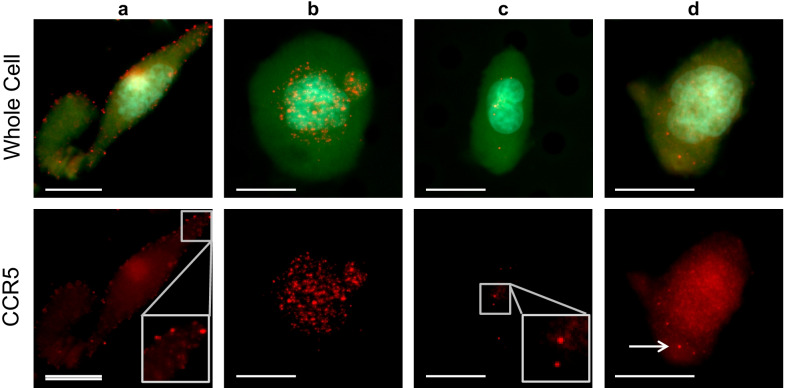
CCR5 Signal Patterns within the Circulating Cells of Breast Cancer Patients. Four examples of CCR5 expressions in CAMLs are shown with the cytoplasm (green), the nucleus (light blue) and CCR5 pools (red). **a** Example of a cell with most of the CCR5 pools on the periphery of the cell. Enlarged view shows CCR5 pools around the perimeter of cell **b** Example of a cell with high CCR5 pooling that appears translocated to in and around the nucleus. **c** Example of a cell with very few internal CCR5 pools. Enlarged view shows the few clear CCR5 pools. **d** Example of a cell with mostly “diffuse” CCR5 signal which overlaps throughout the cytoplasmic area, including the nucleus, with a small number of CCR5 pools (white arrow). Scale bar is 20 µm

After developing a model for high and low CCR5 expression, as well as the nuclear internalization of the receptor in cell lines, we recruited 54 mBC patients to study CCR5 in TACs circulating in the bloodstream. In contrast with cell lines, we found that CCR5 in both CTCs and CAMLs appear mostly as CCR5 pools when present on the surface of the cell (Figs. 3a-c). However, internalized CCR5 signal also appeared as CCR5 pools, but more often appeared as diffuse signal that generally overlapped with the nucleus (Fig. 3d). In the patients tested (*n* = 54), 70% (*n* = 38) had CCR5 positive CAMLs and 41% (*n* = 22) had CCR5 positive CTCs, with positivity defined as ≥ 1 pool. We also found that when CCR5 is present on TACs, it is predominantly located on the surface of the cell. Moreover, we found that with CCR5 + CAMLs, 73% of the CCR5 signal on the cell surface and 20% being internal, with 7% pools appearing translocated to the nucleus. In CTCs, 69% of the CCR5 signal was on the cell surface, 23% was internal, and 8% of pools were translocated to the nucleus (Additional file 1: Fig. S4). Patients that had no TACs also had no CCR5 pools according to our analysis.

## Clinical relevance of CCR5 in breast cancer

**Fig. 4:**
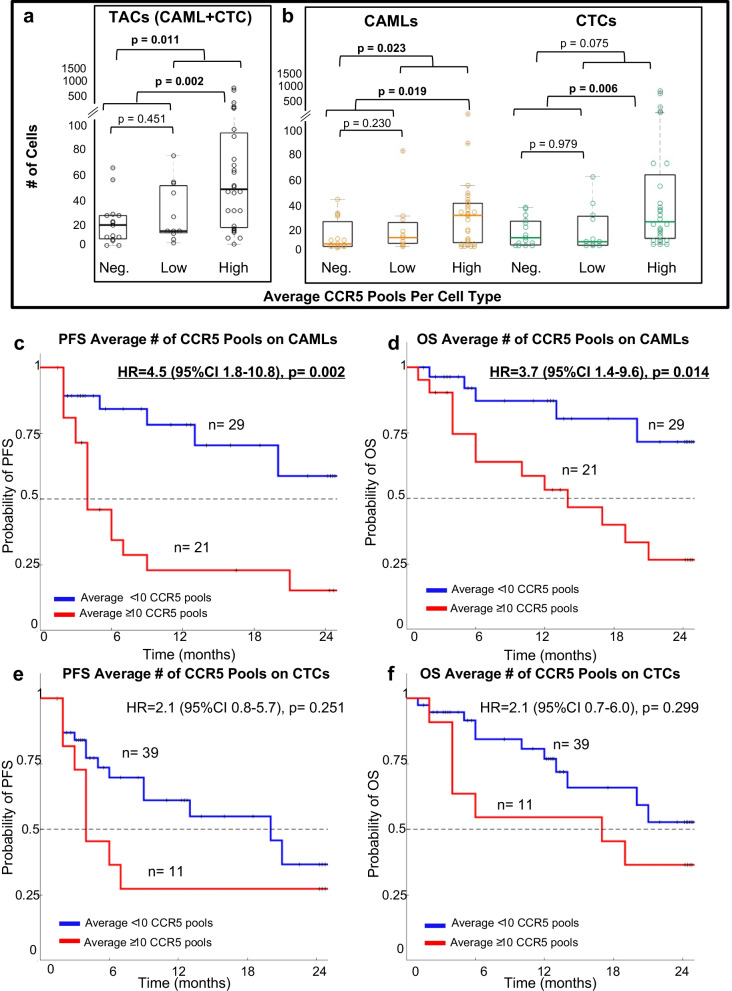
Presence of CCR5 pools in Breast Cancer Patients and Their Clinical Significance. **a** Number of TACs in blood compared to average number of CCR5 pools in the cells. Patients with more TACs had a significantly higher average number of CCR5 pools. CCR5 expression: Negative = 0 pools, Low = 1–9 pools and High = 10 + pools. **b** Relationship of average number of CCR5 pools for CTCs and CAMLs with total number of these cells in the patient. (**c** and **d**) Kaplan–Meier analysis stratifying patients based on their average number of CCR5 pools in CAMLs. (**e** and **f**) Kaplan–Meier analysis stratifying patients based on their average number of CCR5 pools in CTCs

To compare if the motility marker CCR5 was associated with higher tumor-associated cell prevalence in the circulation, we compared patients with high average CCR5 pools against the prevalence of TACs (CTCs or CAMLs) in the blood. Even without subtyping CCR5 expression into negative, low, and high; CCR5 on TACs and number of TACs were independent of one another according to single-factor ANOVA (*p* < 0.05). However, with subtyping, it was determined that patients with negative or low average numbers of CCR5 pools also had fewer TACs in their blood (Fig. 4a). In comparison, patients with high average numbers of CCR5 pools had a significant twofold increase in numbers of both CTCs and CAMLs (Fig. 4b). Patients with no CCR5 pools (*n* = 15) averaged only 14 TACs, while patients with low CCR5 (1–9 pools, *n* = 11) averaged 28 TACs, and patients with high CCR5 (≥ 10 pools, *n* = 28) averaged 51 TACs. While there was no significant difference in number of TACs between patients with negative and low number of CCR5 pools, there was a significant increase in TACs in patients presenting with average ≥ 10 CCR5 pools (*p* = 0.002 for TACs, *p* = 0.019 for CAMLs, and *p* = 0.006 for CTCs) (Figs. 4a and 4b). There was also a significant increase in TACs and CAMLs when comparing patients with 0 total CCR5 pools versus patients with average ≥ 1 CCR5 pool (*p* = 0.011, *p* = 0.023) (Fig. 4a and b). This correlation between upregulated CCR5 signaling and larger populations of motile TACs in the bloodstream suggests that CCR5 could enhance the motility of more aggressive and invasive cells like TACs.

Since the average number of CCR5 pools on TACs appears to relate to an increase in the number of TACs, we compared CCR5 expression with any clinical relationship to progression and survival outcome of the patients. We separately compared patients with high CCR5 pools in CAMLs or CTCs. Patients with an average number of CCR5 pools (calculated as the total number of CCR5 pools found in a patient divided by the number of cells of interest per patient) ≥ 10 in CAMLs were found to have significantly poorer PFS and OS (Figs. 4c and 4d), while an average number of ≥ 10 CCR5 pools in CTCs only non-significantly trended toward worse outcomes (Figs. 4e and 4f). Further, location of CCR5 in TACs (i.e., external surface CCR5 signal, internal CCR5 and nuclear translocated CCR5) had no effect on prognosticating outcomes, as few patients presented with internalized CCR5 signal. Overall, it was determined that the presence of an average number ≥ 10 CCR5 pools on CAMLs was a highly significant predictor of worse PFS and OS (PFS HR = 4.5, CI95% 1.8–10.8, *p* = 0.0021 and OS HR = 3.7, CI 95% 1.4–9.6, *p* = 0.0137).

In CTCs, while an average of ≥ 10 CCR5 pools was not a good cutoff in stratifying patient outcomes (Figs. 4e-f), it was determined that a threshold of an average of ≤ 5 CCR5 pools was approaching significance as an indicator of worse PFS, but not OS (PFS HR = 2.5, CI 95% 1.0–6.2, *p* = 0.0800 and OS HR = 2.1, CI 95% 0.8- 5.5, *p* = 0.2045) (Additional file 1: Figs. S5e-f).

## Discussion

By evaluating the effect of RANTES on the MDA-MB-231 model BC cell line, we visualized patterns of CCR5 signaling (i.e., CCR5 pools, activation, and internalization) that are a mechanistic explanation for the CCR5 signaling present in primary biopsies and circulating TACs in breast cancer patients. We specifically evaluated the MDA-MB-231 cells to compare both hyperploidy PGCCs (large cells) and normal ploidy (small cells) in CCR5 activation/expression, as it has been suggested that PGCCs serve as a model for CAMLs while the normal ploidy MDA-MB-231 cells have been well established as a model for CTCs [19, 30, 40]. After separating the two MDA-MB-231 cell ploidy populations, we determined that no significant difference in CCR5 signaling patterns was detectable. This may be explained by the fact that both PGCCs and normal ploidy cells are part of the same breast cancer cell population, and irregardless of their differing nucleation, these cell types expressed CCR5 in a similar fashion. To further our assertion that CCR5 expression is upregulated with the addition of a ligand (i.e., RANTES), we compared the effect of RANTES on CCR5 expression upregulation both overall on MDA-MB-231 cells and specifically within the nucleus. While this experiment was confirmation of a number of previous studies [6, 13], describing the ligand-dependent activation of CCR5 in response to RANTES, we further verified the endocytic internalization pathway via LAMP1 co-localization staining. In order to confirm that these phenomena are not cell-line specific, we further examined three other breast cancer cell lines (SK-BR-3, Hs 578 T, and MCF-7) with varying hormone statuses to confirm uniformity of CCR5 expression irregardless of hormone status (Additional file 1: Fig. S6). We compared our cell line models to patient samples, showing a similar endocytic pathway for CCR5 internalization. This ligand-dependent activation of cell surface CCR5 in patients suggests that future therapies could be designed to target and inhibit this activation step in order to reduce the motility of those cells and downstream metastasis of disease^3^, though future studies are clearly needed.

It has been shown that CCR5 signaling and the CCR5/RANTES axis in breast cancer is more upregulated in patients with more aggressive disease subtypes, though this upregulation is not commonly seen in primary breast tissue but rather in secondary sites (Additional file 1: Fig. S7) [2]. In this study, while only 15 paired primary tumor biopsies were available, our results demonstrate that CCR5 was negative in 87% (*n* = 13/15) of primary tumor tissue and CCR5 identifiable in only 13% (*n* = 2/15) of patients (Additional file 1: Fig. S7). Further, this positivity was only found in limited areas of the biopsy, which appears as low/weak heterogeneous CCR5 signal (Additional file 1: Fig. S7). This result is not surprising, as CCR5 allows for the mobilization of cells and has been hypothesized that CCR5 presence is mainly present on actively motile cells such as CTCs or tumor macrophages [22, 24], but is downregulated in stagnate fixed cells found at tumor masses [2]. We therefore evaluated actively moving cancer cells (TACs) to quantify the presence of CCR5 in circulation to compare it to cancer progression. In confocal imaging, we visualized CCR5 with clear localization on the surface and within TACs (Additional file 1: Fig. S8). Additionally, we have imaged CCR5 in CAMLs co-stained with CD45 to show localized overlap between these two surface stains (Additional file 1: Fig. S9). In this study, we compared both CTCs and CAMLs as both cell types have been shown to be important in the spread of breast cancer and in predicting clinical outcomes (Additional file 1: Fig. S10).

TACs isolated from the bloodstream were visualized with CCR5 pools and nuclear internalization (Fig. 3). We visualized CCR5 via the standard immunofluorescence assay but in larger future studies we hope to utilize droplet digital PCR (ddPCR), subcellular fractional assays, Western blot assays, and fluorescence-activated cell sorting (FACS) to better evaluate the genomic and proteomic profiling. The diffuse internal CCR5 signaling is likely due to the process of the previously described receptor–ligand complexes being resensitized in the perinuclear space prior to recycling to the cell surface which involved the dissolution of the CCR5 pools (Fig. 3d) [13]. In multiple instances, we observed CCR5 pools localized to the nuclear region of CAMLs, but did not observe this translocation event in CTCs. Since the CCR5 in the perinuclear space was seen as either pools (Fig. 3b) or diffuse signal (Fig. 3d), it posed a challenge to quantify internalized CCR5. As a result, we chose to not specify the localization of the pools within the cell, but rather looked at total number of CCR5 pools. However, the lack of nuclear co-localization in CTCs and the clear co-localization to the nucleus in CAMLs presents an interesting finding in the context of the tumor macrophage/tumor cell axis. Condelis et al. have suggested that mobile tumor cells do not move independently into circulation during metastatic spread, but that the process of CTC movement into the circulatory system is driven by tumor-associated macrophages (TAMs) [23]. In their analysis, it was found that motile macrophages bind to tumor cells at primary tumors and the TAMs then move into circulation via transendothelial migration, pulling tumor cells with them. Furthermore, CAMLs have been shown to exhibit proangiogenic markers and may act as “soil” cells in metastatic cancer spread [22, 23, 35]. This proangiogenic activity of CAMLs enhances the vascularization of tumors and could allow for soil-and-seed CAMLs and CTCs to enhance the spread of aggressive disease [22, 24, 38]. In addition, it has been shown in human BC, a high density of tumor-associated macrophages is correlated with poor prognosis [22, 23]. Here we find that CAMLs in the circulation of mBC patients have the hallmarks of CCR5 activation indicating the possible migratory pathway of TAM entry into circulation.

## Conclusions

The clinical significance of CCR5 signaling on or in CTCs, CAMLs and in cancer cells within tissue biopsies as it relates to survival suggests a possible prognostic and predictive biomarker that may be relevant to assessing patient response. In this preliminary study, we did not have a previous established known cutoff regarding CCR5 in TACs and thus sought to examine whether any clinical significant threshold exists in the data. These data suggest that ≥ 10 CCR5 pools in CAMLs both indicate patients with poorer clinical outcomes, including faster progression and faster death. Though limited by this study’s 54 patient population, the multivariate analysis conducted on the clinically significant variables (Additional file 1: Figs. S10 and S11) found that CAMLs with CCR5 ≥ 10 dots are the most significant independent clinical variable for OS, but not for PFS, and triple-negative status was the only independent significant variable for PFS**.** However, the ability to quantify clinically relevant CCR5 signal in motile cells in patients may have important indications in a number of ongoing early phase clinical trials implementing anti-CCR5 therapeutic drugs. Due to the lack of CCR5 expression in patient biopsies [2] (Additional file 1: Fig. S4), the finding of CCR5 pools in mobile circulating cells (i.e., CTCs and CAMLs) is of great interest in drug-targetable therapies. As TACs are responsible for cancer spread, patients with higher quantities of drug-targetable CCR5 may see great benefit from anti-CCR5 therapy by minimizing the spread of CTCs and/or CAMLs [3, 9, 41]. There are a number of anti-CCR5 drugs that exist in the market, including a number of CCR5 inhibitors in early phase clinical trials, i.e., leronlimab, aplaviroc, vicriviroc, and maraviroc, all of which block the CCR5 receptor and inhibit activation of the pathway [3]. As there are currently a number of ongoing clinical trials that are looking into the efficacy of anti-CCR5 therapies in a variety of different cancers (i.e., breast, colorectal, pancreatic, and non-Hodgkin lymphoma) [3, 9], the addition of a companion diagnostic that identifies CCR5 expression may be beneficial, while in this study we did not evaluate every possible mechanism for the occurrence of CCR5 pooling, including the possibility that CCR5 pooling may be caused by an unknown clinical pathway other than CCR5 activation. However, this study was able to replicate the findings of prior publications by producing CCR5 pools in cell lines, but then further identify a possible clinical association of CCR5 pools in patient’s blood cells which appear to correlate with significantly worse clinical outcomes. While larger clinical studies are now needed to validate both the presence of CCR5 pools in TACs as well as the CCR5 pools association with poorer outcomes, these results do suggest that CCR5 pools may have clinically relevancy. In this initial pilot study, the lack of uniformity in the patient population, the heterogeneity in therapies, and variety in number of prior therapies may likely impact the results and conclusions, which should be accounted for in follow up studies. While preliminary, and requiring validation in further studies, these results suggest that quantifying CCR5 signal on circulating TACs may identify patients who might respond to anti-CCR5 therapy, thus minimizing cancer spread and improving patient survival.

## Supplementary Information

**Additional file1: Fig. S1** MDA-MB-231 and RANTES Bioassay Plate Design. **Fig. S2 **Co-localization of CCR5, RANTES, and LAMP1 in MDA-MB-231 cells or LAMP1 and CCR5 in breast cancer patients. **Fig. S3** Comparing PGCC (Large Cells) versus normal sized cells in MDA-MB-231 cells. **Fig. S4** CCR5 Signal Intensity and Localization in CAMLs and CTCs in 54 BC Patients Randomized **Fig. S5** Kaplan–Meiers of CAMLs and CTCs at alternative thresholds. **Fig. S6** CCR5 Expression in the MDA-MB-231, SK-BR-3, Hs578T and MCF-7 cell lines.. **Fig**. **S7** Primary Biopsy samples stained with CCR5. **Fig**. **S8** CCR5 Confocal Image. **Fig. S9** CCR5 with surface co-staining CD45. **Fig. S10** Multivariate Analysis of Clinical Variables that impact Progression Free Survival (PFS). **Fig**. **S11** Kaplan–Meiers based on CTC number.

## Data Availability

The authors declare that all relevant data supporting the findings of this study are available within the paper and its supplementary information files and any additional raw numerical data are available upon reasonable request.

## Abbreviations

TACs: Tumor-associated cells
CTCs: Circulating tumor cells
CAMLs: Cancer-associated macrophage-like cells
BC: Breast cancer
mBC: Metastatic breast cancer
TAMs: Tumor-associated macrophages
PGCC: Polyploid giant cancer cells
PFS: Progression-free survival
OS: Overall survival
