# Supplementary material for: CCR5 activation and endocytosis in circulating tumor-derived cells isolated from the blood of breast cancer patients provide information about clinical outcome
Source: Breast Cancer Res. 2022 May 23;24:35. doi: 10.1186/s13058-022-01528-w (PMC9125938; doi:10.1186/s13058-022-01528-w)
Supplement: Supplementary file 1 — Additional file1: Fig. S1 MDA-MB-231 and RANTES Bioassay Plate Design. Fig. S2 Co-localization of CCR5, RANTES, and LAMP1 in MDA-MB-231 cells or LAMP1 and CCR5 in breast cancer patients. Fig. S3 Comparing PGCC (Large Cells) versus normal sized cells in MDA-MB-231 cells. Fig. S4 CCR5 Signal Intensity and Localization in CAMLs and CTCs in 54 BC Patients Randomized Fig. S5 Kaplan–Meiers of CAMLs and CTCs at alternative thresholds. Fig. S6 CCR5 Expression in the MDA-MB-231, SK-BR-3, Hs578T and MCF-7 cell lines.. Fig. S7 Primary Biopsy samples stained with CCR5. Fig. S8 CCR5 Confocal Image. Fig. S9 CCR5 with surface co-staining CD45. Fig. S10 Multivariate Analysis of Clinical Variables that impact Progression Free Survival (PFS). Fig. S11 Kaplan–Meiers based on CTC number. [file 13058_2022_1528_MOESM1_ESM.pdf]

	Control Wells	Tagged RANTES (tRANTES)	Untagged RANTES (utRANTES)	
**Large Cells** (PGCCs)	a<br>Big Cells<br>No RANTES	b<br>Big Cells<br>tRANTES	c<br>Big Cells<br>utRANTES	Empty
**Small Cells**	d<br>Small Cells<br>No RANTES	e<br>Small Cells<br>tRANTES	f<br>Small Cells<br>utRANTES	Empty

Supplementary Figure 1. MDA-MB-231 and RANTES Bioassay Plate Design.
(**a** and **d**) served as negative control wells with no RANTES added. (**b** and **e**) Tagged RANTES (125nM) was added to the wells. (**c** and **f**) Untagged RANTES (125nM) was added to the wells.

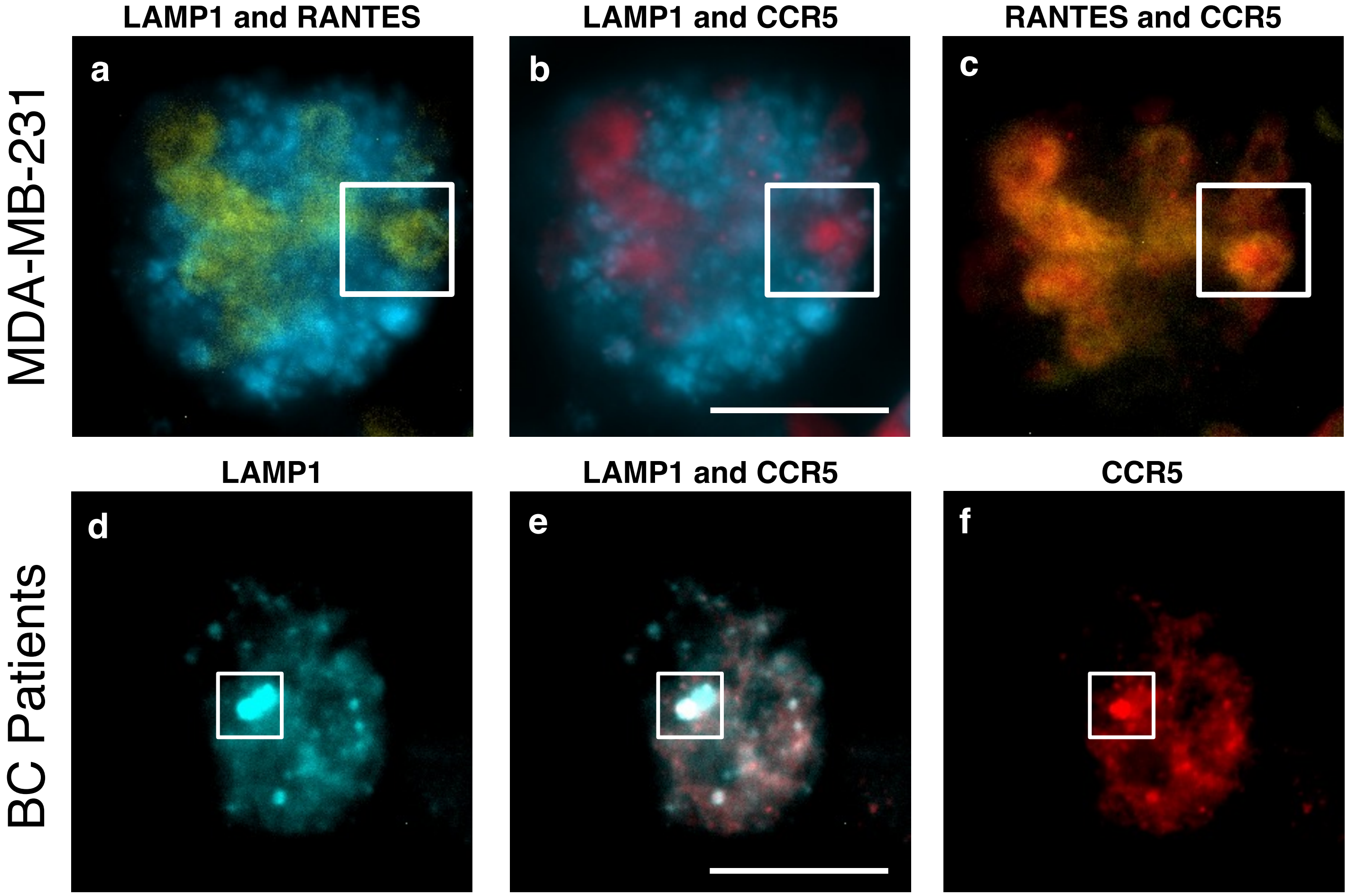

Supplementary Figure 2. Co-localization of CCR5, RANTES, and LAMP1 in MDA-MB-231 cells or LAMP1 and CCR5 in breast cancer patients.

(**a**) Overlapping of RANTES (yellow) and the endosome marker LAMP1 (light blue). (**b**) Overlapping of CCR5 (red) with LAMP1. (**c**) Overlapping of CCR5 (red) and RANTES (yellow). (**a-c**) White box highlights an endosome positive for LAMP1, CCR5, and RANTES. (**d**) LAMP1 highlights endosomes in a patient's CAMLs. (**e**) LAMP1 and CCR5 can be found in separate parts of the cell or co-localized in a single endosome. (**f**) CCR5 pools. (**d-f**) White box highlights co-localized CCR5 and LAMP1, confirming CCR5 endosomal international. Scale bar is 20 μm.

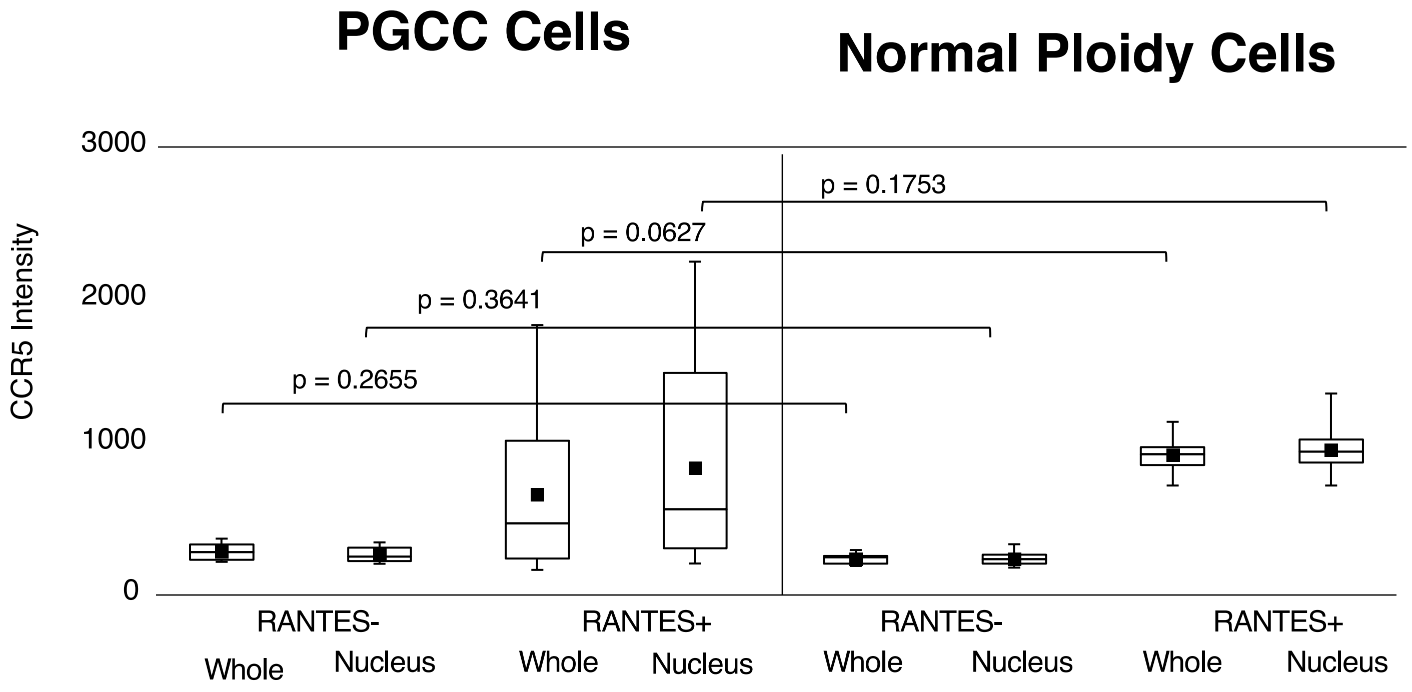

Supplementary Figure 3. Comparing PGCC (Large Cells) versus normal sized cells in MDA-MB-231 cells. When comparing large PGCC (>30 µm) cells (top) and normal sized cells (bottom) without RANTES addition (RANTES-) and with RANTES addition (RANTES+), there was no significant difference between the CCR5 expression in cell populations either in the nucleus or overall.

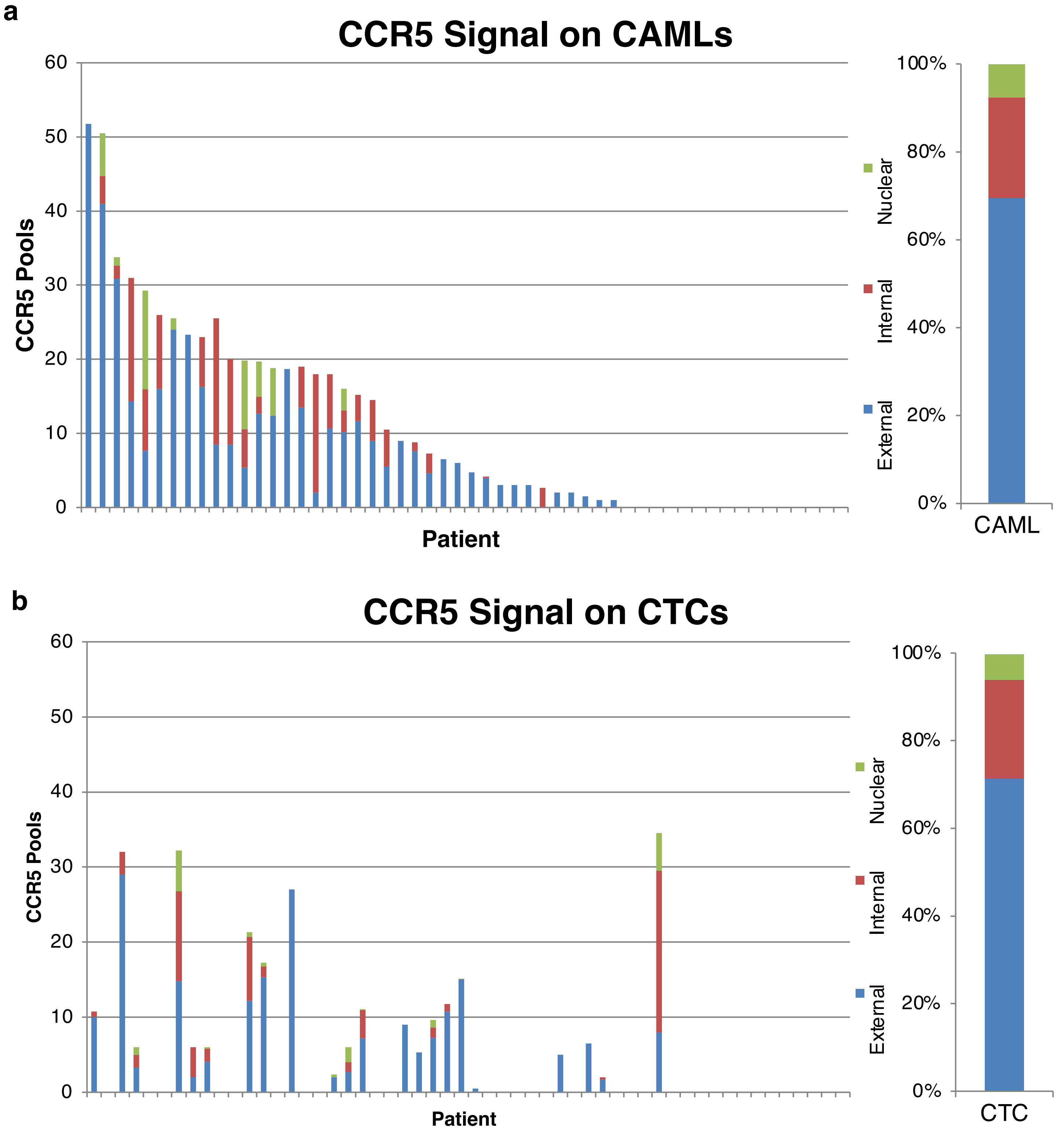

Supplementary Figure 4. **CCR5 Signal Intensity and Localization in CAMLs and CTCs in 54 BC Patients Randomized**

(**a**) In CAMLs, CCR5 signal is predominantly located externally on the surface of the cell (blue), with some internal CCR5 (red) or nuclear (green). 16 patients had 0 CCR5+ CAMLs. (**b**) In CTCs, CCR5 signal was also predominantly located externally on the surface of the cell (blue). 32 patients had 0 CCR5+ CTCs. Overall CCR5 location on the cell was similar between CAMLs and CTCs.

.

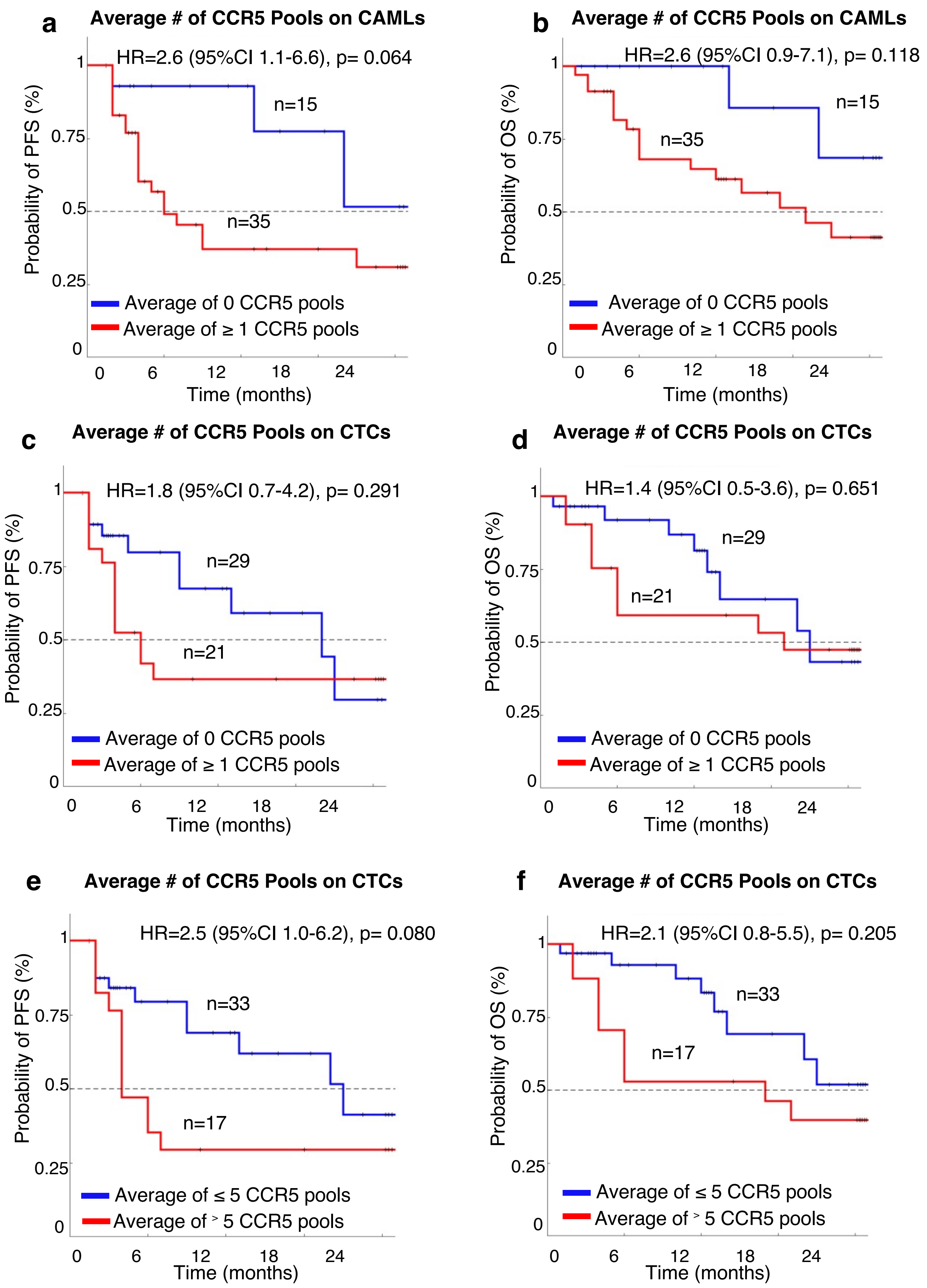

Supplementary Figure 5. **Kaplan Meiers of CAMLs and CTCs at alternative thresholds.** (**a** and **b**) A threshold of 0 total CCR5 pools in CAMLs was not significant for PFS or OS. (**c** and **d**) In CTCs, a threshold of 0 total CCR5 pools was not significant for PFS or OS. (**e** and **f**) In CTCs, a threshold of ≥5 total CCR5 pools was approaching significance for PFS, but not significant for OS.

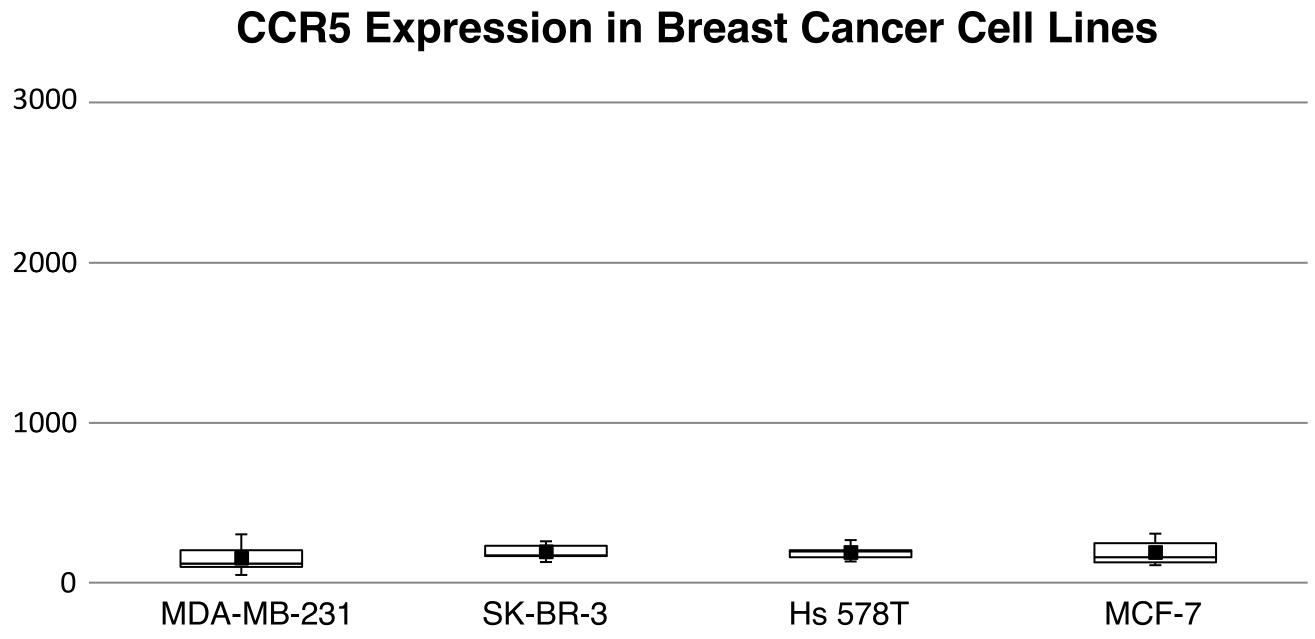

Supplementary Figure 6. CCR5 Expression in the MDA-MB-231, SK-BR-3, Hs578T and MCF-7 cell lines.

In various breast cancer cell lines, net CCR5 expression (overall CCR5 signal minus background signal) is low without the addition of RANTES. MDA-MB-231 and Hs 578T cell lines are both triple negative breast cancer cell lines, while the MCF-7 cell line is estrogen receptor and progesterone receptor positive and the SK-BR-3 cell line is HER2 positive.

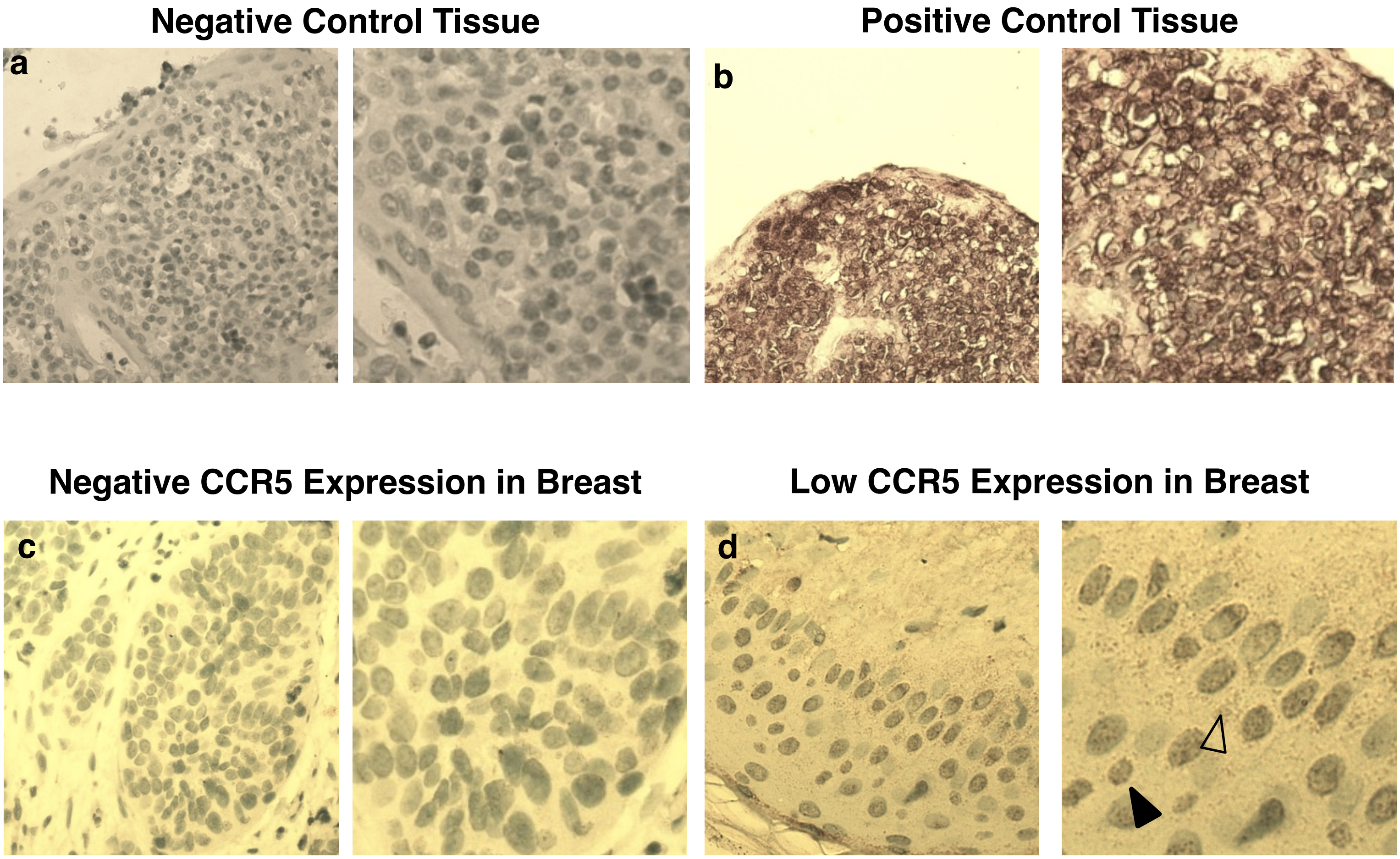

Supplementary Figure 7. Primary Biopsy samples stained with CCR5. (**a**) Negative control tissue with no CCR5 stain. (**b**) Positive control tissue with high CCR5 signal (brown stain). (**c**) In breast cancer biopsies most patient samples were negative (n=13/15). (**d**) In n=2/15, faint CCR5 signal was found in either the cytoplasm of the cell (open arrow) and/or within the nuclear area (black arrow).

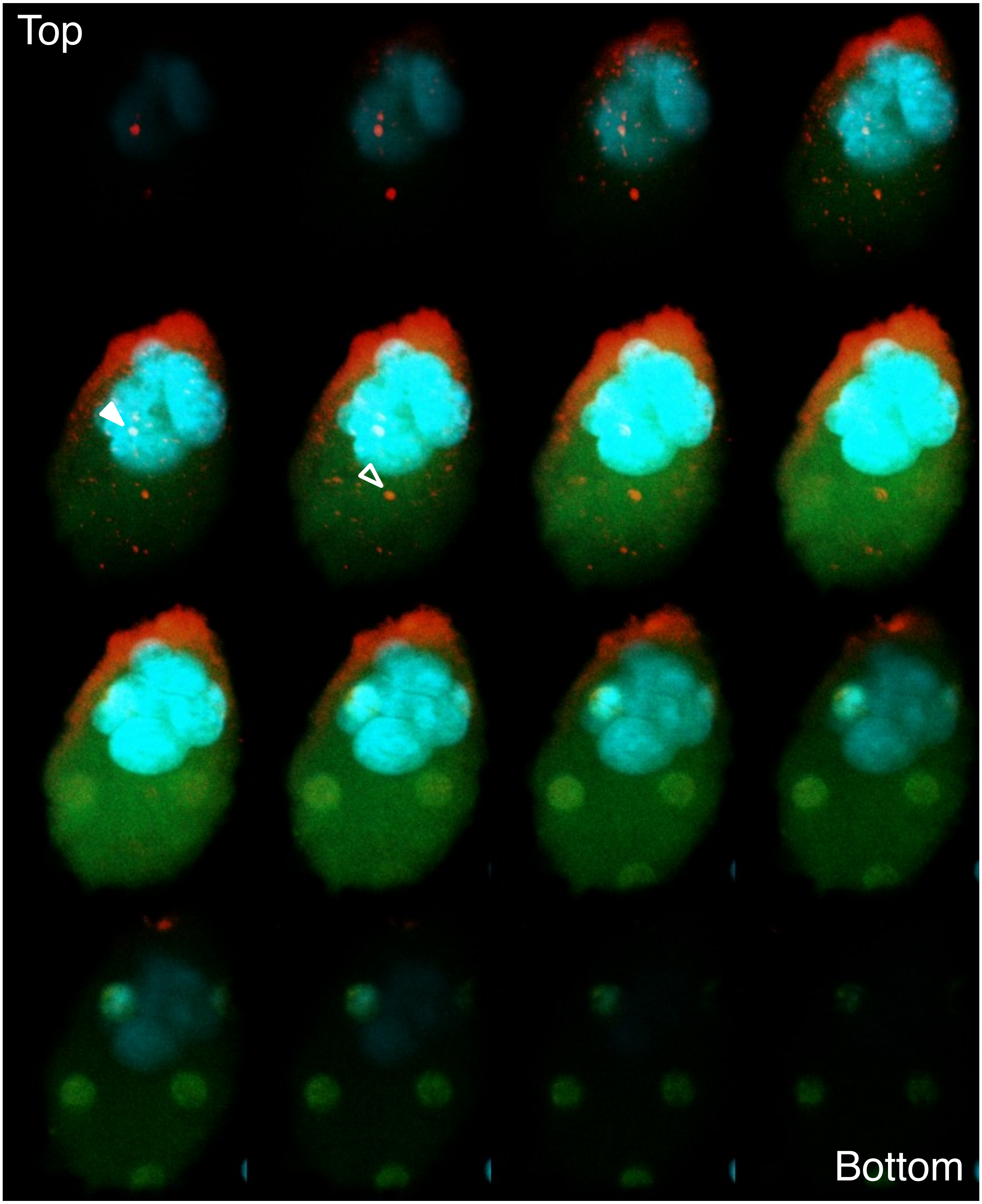

Supplementary Figure 8. **CCR5 Confocal Image.** Confocal images of a CCR5 in a CAML (top to bottom of cell). Consecutive slices of a CAML that has external and internalized CCR5 signal (red), top of cell to bottom. Internalized CCR5 pools were seen within the cytoplasm of the in the first 8 slices (open arrow) and within the nucleus (closed arrow). The upper left portion of the cell had strong diffuse polarized cell surface CCR5 signal, visible in slices 5-12.

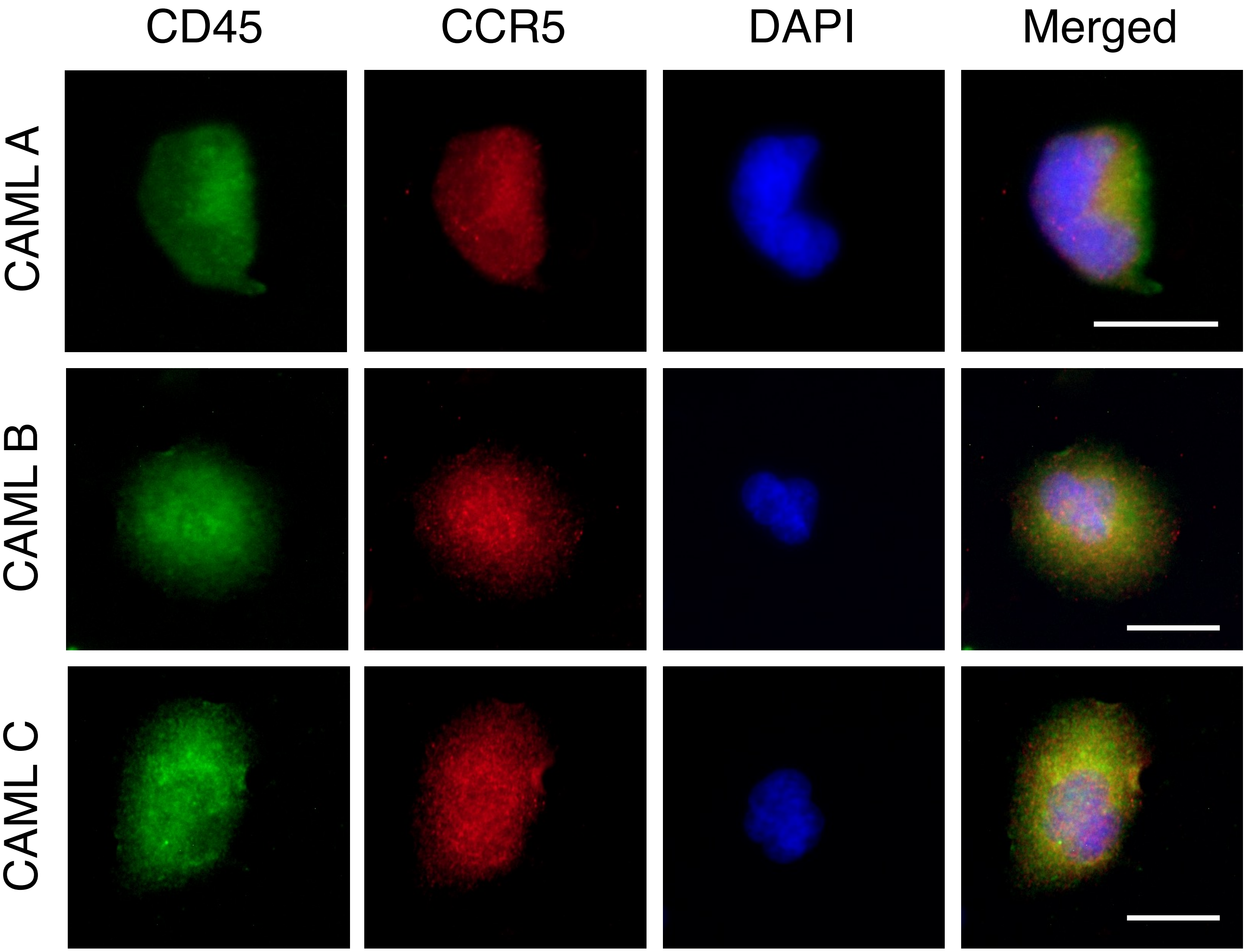

Supplementary Figure 9. CCR5 with surface co-staining CD45. Three CAMLs **(a)**, **(b)**, and **(c)** visualized with CD45 (green), CCR5 (red) and a DAPI-stained nucleus (dark blue)**.** In the merged images, it is clear that CCR5 stain and CD45 overlap consistently. Scale bar is 20 µm.

Characteristic	No. of Patients, n	Patients progressed, n (%)	*p* Value
**CCR5 Pools on CAMLs**			**0.0275**
> 10 CCR5 pools	31	18 (58%)	
< 10 CCR5 pools	23	8 (35%)	
**Lymph Node Spread**			**0.3403**
Yes	21	10 (48%)	
No	26	9 (35%)	
Unknown	7	0 (0%)	
**Brain Metastasis**			**0.2545**
Yes	11	9 (82%)	
No	40	17 (43%)	
Unknown	3	0 (0%)	
**Progesterone Receptor Status**			**0.0574**
Positive	13	6 (46%)	
Negative	37	19 (51%)	
Unknown	4	1 (25%)	
**HER2 Receptor Status**			**0.6775**
Positive	11	6 (55%)	
Negative	40	19 (48%)	
Unknown	3	1 (33%)	

Supplementary Figure 10. Multivariate Analysis of Clinical Variables that impact Progression Free Survival (PFS). CCR5 expression on CAMLs, lymph node spread, brain metastases, progesterone receptor status, and HER2 status were all significant clinical variables in univariate analysis. In a multivariate analysis CCR5 expression on CAMLs was the only significantly independent clinical variable for PFS.

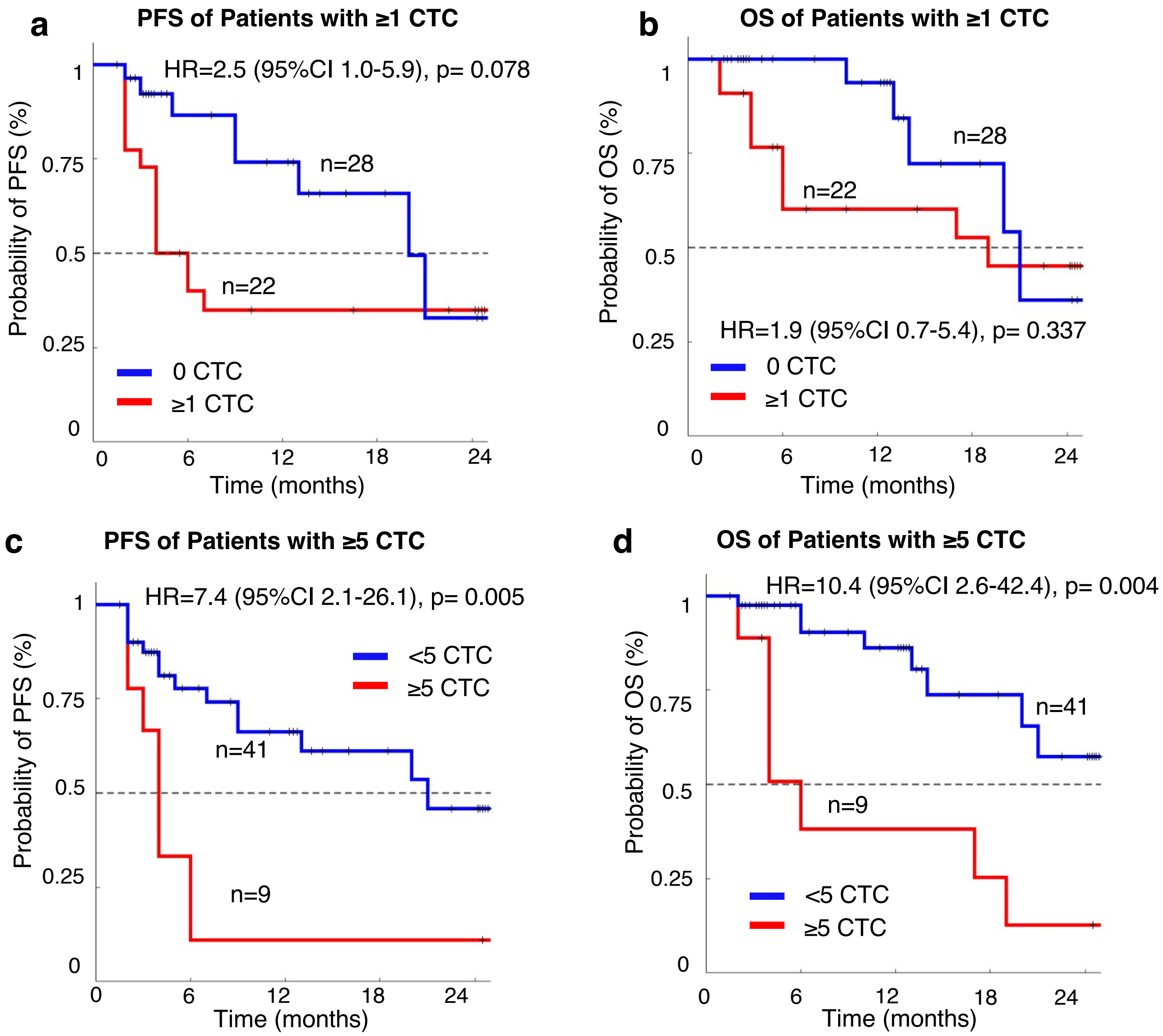

Supplementary Figure 11. **Kaplan Meiers based on CTC number.** (**a** and **b**) PFS and OS based a threshold of ≥1 CTCs. (**c** and **d**) PFS and OS based threshold of ≥5 CTCs.
